# Supplementary material for: Fasciola gigantica, F. hepatica and Fasciola intermediate forms: geometric morphometrics and an artificial neural network to help morphological identification
Source: PeerJ. 2020 Feb 18;8:e8597. doi: 10.7717/peerj.8597 (PMC7034386; doi:10.7717/peerj.8597)

F. gigantica - Cattle - Departures of observed means from the total mean computed over various studies, including ours

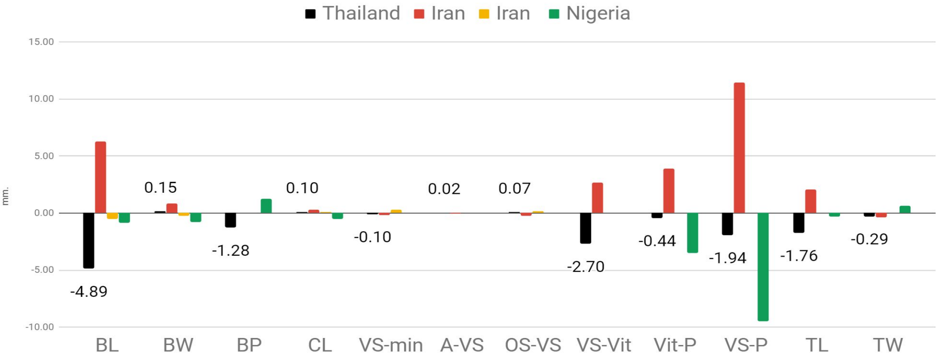

F. hepatica - Sheep - Departures of observed means from the average mean computed over various studies, including ours (black).

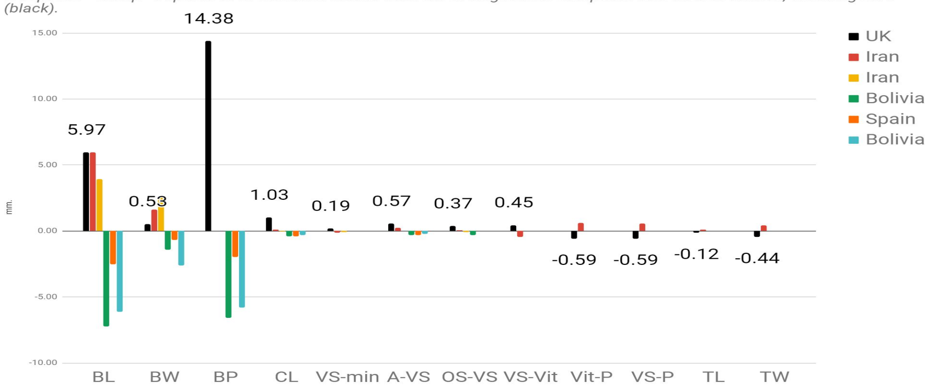

F. intermediate form - Departures of observed means from the average mean computed over various studies, including ours (black).

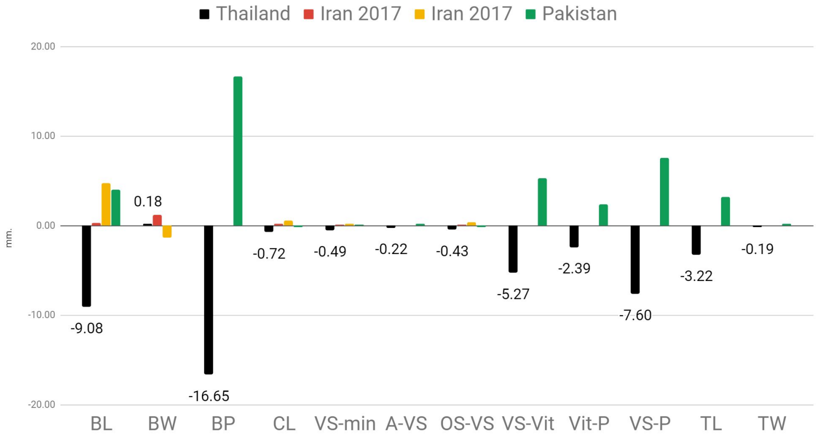

Supplement: Figure S1 — Data were compared graphically with previously published ones, showing the departures of observed means (published ones and ours) from the total means [file peerj-08-8597-s001.pdf]
