# Supplementary material for: Fasciola gigantica, F. hepatica and Fasciola intermediate forms: geometric morphometrics and an artificial neural network to help morphological identification
Source: PeerJ. 2020 Feb 18;8:e8597. doi: 10.7717/peerj.8597 (PMC7034386; doi:10.7717/peerj.8597)
Supplement: Figure S3 — Lane 1: 100 bp DNA ladder, lane 2 to 3: F. gigantica, lane 4: Fasciola intermediate form, lane 5 to 14: F. gigantica, lane 15: Fasciola intermediate form. The PCR digested amplicons were run on 2% agarose gel at 50 V for 180 min. [file peerj-08-8597-s003.pdf]

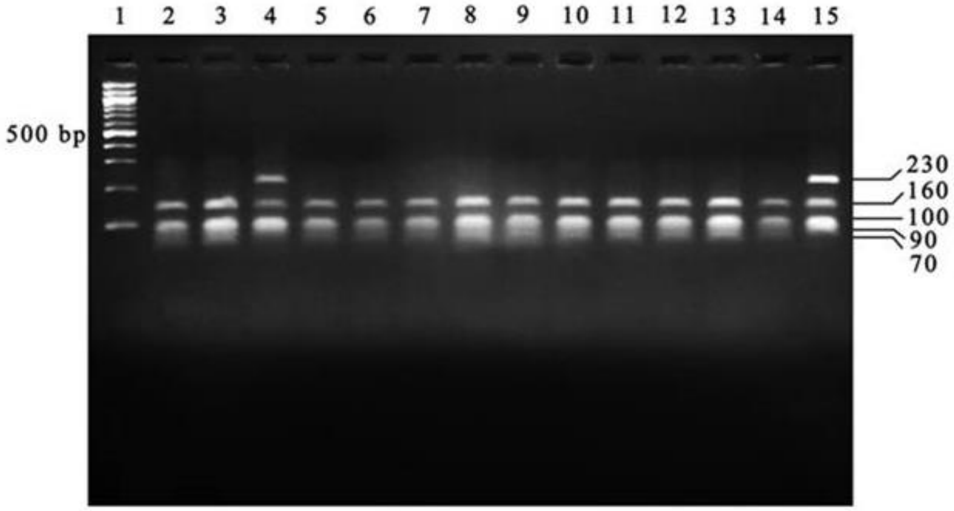

PCR-RFLP patterns of ITS2 amplicon (550 bp) digested by *Nla*III enzyme. Lane 1: 100 bp DNA ladder, lane 2 to 3: *F. gigantica*, lane 4: *Fasciola* intermediate form, lane 5 to 14: *F. gigantica*, lane 15: *Fasciola* intermediate form. The PCR digested amplicons were run on 2% agarose gel at 50 V for 180 min.
