# Supplementary material for: Fasciola gigantica, F. hepatica and Fasciola intermediate forms: geometric morphometrics and an artificial neural network to help morphological identification
Source: PeerJ. 2020 Feb 18;8:e8597. doi: 10.7717/peerj.8597 (PMC7034386; doi:10.7717/peerj.8597)
Supplement: Data S1 [file peerj-08-8597-s005.docx]

**Supplementary data 1: Classification algorithm**

**1/ Validated classification (leave-one out method)**

The classification method, either based on the Mahalanobis distances or on the maximum likelihood, was applied to the sample from which one individual was removed, and the resulting model was applied to classify the removed individual.

Such process was repeated as many times as the number of individuals in the total sample (36), and the final “accuracy” became the number of correctly assigned individuals. This “jack-knife” procedure allows to estimate the confidence which can be given to the classification power of the dataset.

In summary, the seven steps of one classification session were the following:

1/ X, the order number of the individual which is removed (“X” going from 1 to “N”, the total number of individuals)

2/ testing set = individual X

3/ training set = total sample minus individual X,

4/ classification method applied to the training sample obtained step 3/, according to known subdivision into groups

5/ assignation of individual X to one of the groups

6/ X = X+1

7/ if X <N+1, goto step 2/, otherwise exit

**2/ Classification algorithm using the MLP**

As for the general algorithm described above (validated classification), the multilayer perceptron (MLP) was performed on the sample from which one individual was removed, and the resulting “weights” used to classify this individual. Thus, as for the general algorithm, the classification process was repeated as many times as the number of individuals in the total sample (36).

The difference from the general classification algorithm (as applied to other methods than ANN) was that the classification session was repeated 30 times. Indeed, different training sessions are likely to produce different results, and we used as the final result the average (and standard deviation) after 30 sessions.

Thus, the classification session as summarized above was repeated 30 times; the average and standard deviations were computed on these 30 scores. For more details about the multilayer perceptron configuration please refer to the **Supplementary data 2**
