# Supplementary material for: Fasciola gigantica, F. hepatica and Fasciola intermediate forms: geometric morphometrics and an artificial neural network to help morphological identification
Source: PeerJ. 2020 Feb 18;8:e8597. doi: 10.7717/peerj.8597 (PMC7034386; doi:10.7717/peerj.8597)
Supplement: Supplemental Information 6 [file peerj-08-8597-s006.docx]

**Supplementary data 2: Multilayer perceptron**

We used a multilayer perceptron trained using the backpropagation algorithm, as developed in library “mlp.js” (Ulf Biallas, see acknowledgements), freely available from <https://www.npmjs.com/package/mlp>

The library uses the plain backpropagation algorithm without any optimization. The whole source code may be found on GitHub <https://github.com/ulfbiallas/npm-mlp>

and the implementation of the backpropagation algorithm can be found in <https://github.com/ulfbiallas/npm-mlp/blob/master/src/>

The “mlp.js” library uses a sigmoid activation function in all layers. Currently, the library does neither support different activation functions for each layer nor custom activation functions. The activation function is hard-coded to f(x) = 1 / (1 + exp(-0.5 * x)).

The “mlp.js” library uses as cost function the mean squared error, based on the difference between the output for a training vector and the true, observed one.

In our study, the output was a three element array (*Fasciola gigantica, F. hepatica, intermediate form*)

[1, 0, 0] for the first species

[0, 1, 0] for the second species

[0, 0, 1] for the last one.

The output for a training vector could be for instance [0.4, 0.3, 0.8]. In that example, the output would point to the third species above because 0.8 is the value closest to 1.

The training set was composed of 8 randomly selected specimens by species. The remaining individuals constituted the validation set.

For “mlp.js” library, available hyperparameters were the number of “hidden layers”, the number of “neurons” in each hidden layer, the “error” (difference between computed and expected outputs) and the “learning rate”. In our application to the classification of *Fasciola sp*., the following configuration was used:

- Number of hidden layers: 1
- Number of neurons by hidden layer: 3
- Learning rate: 0.015
- Error : 0.15

The optimisation was performed through the backpropagation algorithm. The training process was iteratively performed until the error fall under the limit error, initially put by us at 0.15

As indicated by the <https://www.npmjs.com/package/mlp> site, “*... Depending on the training set, the learn rate and the structure of the perceptron, this step may take a while. If necessary, you can add additional exit conditions to the loop (e.g. a maximum number of iterations).”*

We then configured the use of the “mlp” script as follows:

. If the error remains larger than 0.15 after 35000 iterations (epochs), the error hyperparameter is increased by adding 0.15 to the previous value, and the training is continued for a new loop of 35000 epochs.

. If the error falls under 0.15, the weights are applied to the validation set, and saved for later use to the condition that the total score obtained on the validation set was equal or higher than 75%.

. In case the score of 75% was not reached, the training process was continued using however a new training set, and as a consequence a new validation set.

. If the conditions above are obtained, weights are saved and used to assign this individual to species.

. Some exit conditions were added in case the 75% of correct assignation on the validation set could never be reached.

The above steps represent the classification of a single individual, the one which was removed from the data before training.

A complete session was thus the above steps repeated for each individual, i.e. 36 times.

Because two sessions did not necessarily produce exactly the same results, the complete session was repeated 30 times and an average result and its standard deviation (68% of the distribution) was presented (see **Supplementary data 1 “Classification algorithm”)**
